# Successful Aging and Self‐Neglect Among Community‐Dwelling Older People

**DOI:** 10.1111/phn.13439

**Published:** 2024-10-07

**Authors:** Esma Nur Sert, Aysegul Ilgaz

**Affiliations:** ^1^ Faculty of Nursing Akdeniz University Antalya Turkey

**Keywords:** aging, healthy aging, psychosocial support systems, self‐neglect

## Abstract

**Objective:**

It was aimed to explore the relationship between successful aging and self‐neglect and factors affecting successful aging among community‐dwelling older people.

**Methods:**

This cross‐sectional study was conducted with 316 participants aged 60 years and older in a Family Health Center (FHC). The data were collected by using the questionnaire form, Successful Aging Scale and Self‐Neglect Scale. The questionnaire form includes sociodemographic characteristics, independence (Katz Daily Life Activities Scale) and well‐being status (WHO‐5 Well‐Being Index), psychosocial support (Multidimensional Perceived Social Support Scale), and depressive symptoms (two questions‐complaints such as feeling depressed or hopeless and loss of interest and inability to enjoy life).

**Results:**

Participants had a mean age of 67.5 (SD: 6.5) years, 55.4% were female. A strong positive correlation was determined between successful aging score and self–neglect score (*r* = 0.741, *p* < 0.001). Factors affecting successful aging were age, working status, education level, body mass index, physical activity, subjective health perception, health screening status, self‐neglect level, depressive symptoms, independence in activities, well‐being status, and psychosocial support. Gender, income status, chronic disease presence, cigarette and alcohol use, living alone, and family type did not affect it. According to multivariate linear regression, the factors affecting successful aging were age, psychosocial support presence, and self‐neglect level (*p* < 0.05).

**Conclusion:**

Older people have low successful aging and self‐neglect scores. Successful aging is better at a young age, in the presence of psychosocial support and a low level of self‐neglect. It is recommended to increase the existence of psychosocial support for them and to plan interventions to reduce self‐neglect.

## 1 Introduction

The percentage of the old population was 10% worldwide in 2022 (World Bank 2022). In 2023, it was slightly less than 4% in least developed countries and much less than 20% in developed countries. The worldwide older population aged 65 and over is expected to be 27.8% in developed countries and 6.1% in least developed countries by 2050 (UN 2023). In Turkey, the population aged 65 and over has reached 8 million 722 thousand 806 people in 2023. The proportion of older people in the total population increased to 10.2% in 2023 (Turkish Statistical Institute, 2023). According to the World Health Organization, people who reach the age of 60 in developing countries are considered older adults (WHO 2022). In Turkey, a person must be 60 years of age or older to be admitted to a nursing home, and this age group is also known as the older population (Ministry of Family and Social Services 2020).

In both developed and developing countries, with the rapid aging of the population, the prolongation of life expectancy and the increase in life expectancy at birth, healthy and successful aging among older people has become an important issue. The United Nations has officially declared the years 2021–2030 as the “Decade of Healthy Aging” (WHO 2021). Healthy aging requires not only the absence of disease but also the preservation of basic functional abilities throughout life. Successful aging means the absence of diseases and physical and cognitive deficiencies, is characterized by good health, and is positively associated with happiness (Rowe and Kahn 2015; Shojima et al. 2024). To ensure successful aging, we should focus on dimensions such as self‐acceptance, establishing positive relationships with others, and self‐determination, controlling the environment, having a purpose in life, and personal development (Giles et al., 2022). Successful aging requires us to make concessions for things like losses, flaws, limitations, opportunities, and resources already in place (Silagi et al. 2015). Success in aging is more impacted by variables like physical activity level, social interactions, and the attitudes of older people than it is by constants like an individual's genetic condition (Ayers et al. 2014; Dziechciaż and Filip 2014). In addition, literacy is a key component of good health and can play an important role in successful aging. Older people with higher levels of education and health literacy tend to age more successfully (Gomez, Woods, and Beltran‐Najera 2024; Kolcu et al. 2023). Moreover, it is necessary to consider the cultural, social, and religious conditions of each society to ensure successful aging for older people since the concept of successful aging depends on the cultural context of the society (Estebsari et al. 2020). Studies conducted in Korea and Norway have determined that 13%–14% of older people achieve successful aging (Bosnes et al. 2017; Jang 2020).

Factors associated with successful aging are age, gender, education level, economic status, alcohol addiction, smoking, exercise, perception of health status, health screening, cohabitants, satisfaction with spouse and children, communication and contact with family/relative/friend/neighbor frequency, number of close relatives/friends/neighbors, and access to social resources in the community (Jang 2020). Good sexual and reproductive health in middle age and low cardiovascular risk factors (non‐smoking, normal body mass index, normal cholesterol level) are associated with successful aging (Urtamo et al. 2019). In a study conducted across Turkey, chronic diseases such as obesity, hypertension, arthritis and joint diseases, and diabetes emerge in the fifties, physical function capacity decreases with each passing year, and the need for support and care from others in both basic and vehicular life activities increases. For this reason, it is emphasized that there are no positive developments in healthy and successful aging in Turkey (Ministry of Health 2016).

One of the factors associated with successful aging is self‐neglect (İlhan et al. 2020; Özsungur 2020). A self‐neglecting older person is unable to fulfill his/her personal care such as nutrition and medical care, and in this case, successful aging may be adversely affected. Self‐neglect is defined as an inability to take care of oneself with resistance to receiving care from others (Doron, Band‐Winterstein, and Naim 2013). Self‐neglect typically manifests when an individual's peculiar or risky conduct is seen. Older persons and others who care for them might not recognize the seriousness of the changes that signify self‐neglect, though, because these behaviors take time to manifest (Burnett et al. 2014). One review reported that the prevalence rate of self‐neglect among older people living in the community ranged from 18.4% to 29.1% (Yu et al. 2020).

Risk factors for self‐neglect include sociodemographic characteristics (male gender, older age, low economic status, ethnicity, lower education level, marital status, and lower number of children), health‐related characteristics (cognitive impairment, low physical functioning, poor nutritional status, comorbidity, and pain), psychological characteristics (depressive symptoms), and social characteristics (living alone, lower social networks and social participation, poor neighborhood relations) (Yu et al. 2020). Individuals who neglect themselves have more physical dysfunction, poor quality of life, and depressive symptoms than other individuals (İlhan et al. 2020). There are studies in the literature that deal with the concepts of successful aging (Bosnes et al. 2017; Jang 2020; Özsungur 2020) and self‐neglect (Dong, Simon, and Evans 2013; Dong 2014, 2017) separately. Our literature review revealed no study investigating the effect of self‐neglect on successful aging. Thus, this study aimed to explore the successful aging and self‐neglect levels of community‐dwelling older people and to reveal the factors affecting successful aging. Research hypotheses were as follows: (1) self‐neglect of older people is more likely to be associated with successful aging; (2) sociodemographic characteristics, independence and well‐being status, psychosocial support, and depressive symptoms would be associated with successful aging.

## 2 Method

### 2.1 Design and Setting

This cross‐sectional study was prepared in accordance with the STROBE guideline (The Strengthening the Reporting of Observational Studies in Epidemiology [STROBE] Statement: guidelines for reporting observational studies) (Vandenbroucke et al. 2007). Ethical permission was obtained from an ethics committee for this study and institutional permission was obtained to conduct it in the Family Health Center (FHC). Individuals who came to the FHC for any reason (such as routine checks, syringe administration, prescribing medication, having blood drawn, showing blood results, etc.) were asked their age, and if they were over 60 years old, they were asked whether they wanted to participate in our study. Before interviewing those who gave consent, participants were verbally informed about the purpose of the research and the results we aimed to achieve, and written consent forms were given to older people. Written consent was then obtained voluntarily. It was stated that they would withdraw from the research whenever they wanted. Throughout the research, the principles of the Declaration of Helsinki were followed. The research was carried out in a FHC located in a city center in the south of Turkey between November 2021 and May 2022.

### 2.2 Sample

The population of the research consists of individuals aged 60 and over who are registered with FHC. Since the number of individuals aged 60 and over registered with the FHC could not be reached in terms of the security of personal data, the formula (*n* = p.q.t^2^/d^2^) was used in the calculation of the research sample, and the sample size was calculated for the variables of successful aging and self‐neglect. For successful aging, 185 people were calculated using the prevalence (14%) in the study of Bosnes et al. (2017) (*n* = [0.14].[0.86].[1.96]^2^/[0.05]^2^) (Bosnes et al. 2017). By using the prevalence (29%) in a study conducted for self‐neglect (Dong 2014), the sample size was calculated as 316 people and this number was reached at the end of the study (*n* = [(0.29].[0.71].[1.96]^2^/[0.05]^2^). The inclusion criteria were the age of 60 and over, speaking Turkish, having good cognitive status (with orientation to place, time, and person), not having hearing problems, and volunteering to participate in the study. The exclusion criterion was having a self‐reported psychiatric disorder. Convenience sampling was used to select the participants.

### 2.3 Data Collection

The data were collected by the researchers in the FHC, using face‐to‐face interviews with the participants in an average of 20 min. Interviews with older people were conducted in a quiet, well‐lit, well‐ventilated, and suitable environment (in a room of the FHC) where individuals could express themselves comfortably, in accordance with social distancing, mask, and hygiene rules.

### 2.4 Measures

#### 2.4.1 Descriptive Characteristics Form

Age, gender, marital status, educational status, employment status, and perception of income were questioned and height and weight were measured in the FHC. In addition, smoking, alcohol use, exercise status (mild physical activity each week: walking; moderate activity: light loads, normal speed cycling, etc.; vigorous activity: heavy lifting, digging, aerobics, basketball, etc.), presence of chronic disease, health perception (good, moderate, bad), participation in health screenings, family type, presence of children helping the older people, and who they live with were determined.

#### 2.4.2 Successful Aging Scale

The Turkish validity and reliability study of the Successful Aging Scale, developed by Reker (2009), was conducted by Özsungur and Hazer (2017). This scale consists of two sub‐dimensions: healthy lifestyle (3 items) and coping with problems (7 items) and a total of 10 questions. The scale was prepared in a 7‐point Likert type, each item was scored from 1 to 7 (I strongly agree 7, agree 6, partially agree 5, undecided 4, partially disagree 3, disagree 2, and strongly disagree 1). The lowest 10 and the highest 70 points are obtained from the scale, and as the score increases, successful aging status increases. Healthy lifestyle Cronbach's alpha coefficient was determined as 0.80, coping with problems 0.79, and 0.85 for the total scale (Özsungur and Hazer 2017).

#### 2.4.3 IMS Self Neglect Questionnaire (Istanbul Medical Faculty Screening for Elderly Self Neglect)

It was developed in Turkish by İlhan et al. (2020). The self‐neglect questionnaire consists of 11 items related to personal hygiene, health habits, and social functionality. The answer options for these items are “yes” (1 score) or “no” (0 score). The total score is the sum of “yes” answers, 1 point each. The total test score that can be obtained from the scale varies between 0 and 11 points. Lower test scores indicate a higher probability of self‐neglect. A total score of 7 or less reflects self‐neglect among older people. The Cronbach's alpha coefficient for the scale is 0.71 (İlhan et al. 2020).

#### 2.4.4 Katz Daily Living Activities Scale

It was developed by Katz et al. (1963) and adapted into Turkish by Pehlivanoğlu et al. (2018). In this scale, self‐care activities of the individual such as bathing, dressing, toileting, transferring, continence, and feeding are evaluated. If the individual performs the activities of daily living independently, 1 score is given, if he does it with assistance, 0 score; 0–2 score are evaluated as dependent, 3–5 score semi‐dependent, 6 score independent. The Cronbach's alpha coefficient was found to be 0.83 (Pehlivanoğlu et al. 2018).

#### 2.4.5 WHO‐5 Well‐Being Index

This index was validated in 2001 by Bonsignore et al. (2001). Its Turkish validity was made by Erhan Eser in 1999 and its validity and reliability among older people was conducted in 2019 (Eser et al. 1999). The six‐point Likert‐type index includes questions that evaluate how individuals often feel in the last 2 weeks and is scored from 0 (never) to 5 (always). The total scores are summed up, multiplied by 4, and converted to 0–100 points. The higher the score from the index, the better the emotional state. In a study conducted for the reliability and validity of the Turkish version of the scale for use in adults and older people, Cronbach's alpha coefficient was found to be 0.81 for adults and 0.86 for older people (Eser et al. 2019).

#### 2.4.6 Multidimensional Scale of Perceived Social Support

The scale developed by Zimet et al. (1990) was translated into Turkish by Eker and Arkar in 1995 and was revised in 2001 (Eker, Arkar, and Yaldız 2001). There are three sub‐scales (family, friends, and a special person). Although there are four items in each sub‐scale and 12 items in the total scale. The seven‐point Likert scale consists of strongly agree (7), agree (6), slightly agree (5), undecided (4), somewhat disagree (3), disagree (2), strongly disagree (1). The lowest score to be obtained from the whole scale is 12, and the highest score is 84. A high score indicates high perceived social support. In the Turkish adaptation study of this scale, Cronbach's alpha coefficient was found to be 0.89 (Eker, Arkar, and Yaldız 2001).

#### 2.4.7 Depressive Symptoms

Depressive symptoms were evaluated using the two questions recommended by the Ministry of Health in Turkey. These questions are “The state of individuals feeling depressed or hopeless almost every day in the last two weeks” and “the presence of complaints such as loss of interest or not being able to enjoy life.” Those who answered yes to both questions were determined to have depressive symptoms and were referred to a psychiatrist (Ministry of Health 2015).

### 2.5 Data Analysis

IBM Statistical Package for the Social Sciences (SPSS) software version 22.0 was utilized for statistical analysis. Whether there was a normal distribution was evaluated by the Normality test (Kolmogrov–Smirnov test) and the skewness kurtosis coefficient (between − 1.96 and 1.96) (Field 2009). Pearson correlation analysis was used to analyze the relationship between two continuous variables. Intraclass correlation coefficients were defined as good (coefficient of 0.80–1.00), fair (coefficient of 0.60–0.79), bad (coefficient 0.20–0.60), and very bad (0–0.19) (Mukaka 2012). Two groups were compared using an independent‐sample *t*‐test, and three or more groups using a one‐way analysis of variance (ANOVA) with the Bonferroni as post hoc. multivariate linear regression analysis was conducted with successful aging score as the outcome and sociodemographic, health and clinical data as predictors. For these predictors, variables found to be significant in pairwise comparisons (all variables with *p* < 0.05) were included in this regression model. The regression used the “enter” method to examine the significant impact of all variables simultaneously. For statistical significance, the *p* value was established at 0.05.

## 3 Results

The mean age of participants in the study was found to be 67.5 ± 6.5. 55.4% of the participants were female, 57.6% of them were secondary school or higher, 63.6% were married, and 57.3% were retired. Income status was viewed as “income is less than the expenses” by 40.8% of participants. Table 1 provides comprehensive data on sociodemographic and health characteristics and Table 2 shows the clinical data of participants. The Successful Aging Scale mean score was 43.1 ± 8.5, the Self‐Neglect Scale mean score was 6.6 ± 2.0, the Katz Daily Living Activities Scale mean score was 5.6 ± 0.6, the WHO‐5 Well‐Being Index mean score was 39.7 ± 19.7, and Multidimensional Perceived Social Support Scale mean score was 48.3 ± 11.2 (Table 2).

**TABLE 1:** Sociodemographic and health characteristics of older people.

Variables		*n*	%
**Sociodemographic variables**			
Sex	Female	175	55.4
	Male	141	44.6
Education level	Primary school and less	134	42.4
	Secondary school and above	182	57.6
Marital status	Married	201	63.6
	Single	115	36.4
Income status	Income = expenditure and income > expenditure	187	59.2
	Income < expenditure	129	40.8
Working status	Not working	97	30.7
	Working	38	42.7
	Retired	181	57.3
The person who lives	Alone	66	20.9
	With a person (spouse, children, etc.)	250	79.1
Family type	Nuclear family	276	87.3
	Extended family	40	12.7
The presence of children helping older people	Yes	151	47.8
	No	165	52.2
**Health characteristics**			
Presence of chronic disease	Yes	239	75.6
	No	77	24.4
Chronic diseases^a^	Diabetes mellitus	109	34.5
	Hypertension	150	47.5
	Hypercholesterolemia	68	21.5
	CVD	52	16.5
Smoking status	Yes	96	30.4
	No	220	69.6
Alcohol status	Yes	45	14.2
	No	271	85.8
Body mass index	Healthy weight	96	30.5
	Overweight	124	39.4
	Obese	95	30.2
Individual's perception of health	Poor perception	40	12.7
	Moderate perception	145	45.9
	Good perception	131	41.4
Health screening status	Yes	199	63.0
	No	117	37.0

^a^ There is more than one chronic disease among the participants.

**TABLE 2:** Clinical data of older people.

Clinical variables	Mean	SD
The successful aging	43.1	8.5
The self‐neglect score	6.6	2.0
Katz Daily Life Activities Scale	5.6	0.6
WHO‐5 Well‐Being Index	39.7	19.7
Multidimensional Perceived Social Support Scale	48.3	11.2
The number of days of mild physical activity	4.1	2.1
Duration of mild physical activity (min)	175.0	152.2
The number of days of moderate physical activity	1.3	0.6
Duration of moderate physical activity (min)	44.0	23.8
The number of days of vigorous physical activity	2.9	2.3
Duration of vigorous physical activity (min)	171.4	139.3

		*n*	%
The risk of self‐neglect	Yes	201	63.6
	No	115	36.4
Depressive symptoms	Yes	94	29.7
	No	222	70.3
Mild physical activity each week	Yes	160	50.6
	No	156	49.4
Moderate physical activity each week	Yes	40	12.7
	No	276	87.3
Vigorous physical activity each week	Yes	7	2.2
	No	309	97.8

Table 3 examines the sociodemographic and health characteristics that may affect successful aging. Successful aging was affected by education, working status, body mass index, individuals' perception of health, and health screening status. Those whose education level was a secondary school or above and those who had a health screening have a high successful aging score. According to the Bonferroni test, each group was significant. The scores of working individuals compared to non‐working individuals, working individuals compared to retirees, and retired individuals compared to non‐working individuals were found to be higher. The Bonferroni test revealed that those with a normal body mass index had a higher successful aging score than those who were obese. According to the Bonferroni test, those with a good health perception compared to those with a medium health perception, those with a good health perception compared to a bad health perception, and those with a medium health perception compared to a bad health perception had higher successful aging scores. However, no relationship was found between the variables such as gender, marital status, income status, family type, living alone, presence of children helping older people, presence of chronic disease, smoking and alcohol use, and successful aging score (*p* > 0.05) (Table 3).

**TABLE 3:** Sociodemographic and health characteristics affecting the score of successful aging.

		Successful aging score		
Sociodemographic variables		Mean (SD)	*t* ^a^/*f* ^b^	*p* values
Sex	Female	42.6 (8.9)	– 1.08^a^	0.283
	Male	43.6 (8.0)		
Education level	Primary school and less	40.3 (8.0)	– 5.20^a^	**< 0.001**
	Secondary school and above	45.1 (8.3)		
Marital status	Married	43.2 (8.7)	0.46^a^	0.649
	Single	42.8 (8.3)		
Income status	Income = expenditure and income > expenditure	43.7 (8.4)	1.60^a^	0.110
	Income<expenditure	42.1 (8.6)		
Working status	Not working (1)	40.6(7.5)	10.56^b^	**< 0.001**
	Working (2)	47.8(8.6)		
	Retired (3)	43.3(8.6)		
	Post‐hoc ^c^	2 > 1, 2 > 3, 3 > 1		
The person who lives	Alone	43.2 (8.3)	0.19^a^	0.848
	With a person	43.0 (8.6)		
Family type	Nuclear family	43.2 (8.6)	0.82^a^	0.411
	Extended family	42.0 (8.0)		
The presence of children helping older people	Yes	43.6 (8.1)	1.05^a^	0.295
	No	42.6 (8.9)		
		***r***		***p* values**
Age		– 0.272 ^d^		**< 0.001**

Health characteristics		Mean (SD)	*t* ^a^ /*f* ^b^	*p* values
Presence of chronic disease	Yes	42.7 (8.2)	– 1.41^a^	0.162
	No	44.3 (9.4)		
Smoking status	Yes	43.8 (8.7)	0.95^a^	0.345
	No	42.8 (8.4)		
Alcohol status	Yes	45.1 (8.1)	1.73^a^	0.085
	No	42.7 (8.6)		
Body mass index	Healthy weight (1)	44.4(8.5)	3.53^b^	**0.030**
	Overweight (2)	43.4(8.0)		
	Obese (3)	41.2(8.9)		
	Post‐hoc ^c^	1 > 4		
Individual's perception of health	Poor perception (1)	37.4(7.5)	15.33^b^	**< 0.001**
	Moderate perception (2)	42.5(8.1)		
	Good perception (3)	45.4(8.4)		
	Post‐hoc ^c^	3 > 1, 3 > 2, 2 > 1		
Health screening status	Yes	44.4 (7.7)	3.60^a^	**< 0.001**
	No	40.8 (9.3)		

^a^ Independent samples *t*‐test.

^b^ One‐way analysis of variance (ANOVA).

^c^ Bonferroni test was performed in post‐hoc analysis.

^d^ Pearson correlation test.

A highly positive and significant correlation was found between the successful aging score and the self‐neglect score (*r* = 0.741, *p* < 0.001). A low level negative significant correlation was found between successful aging score and age, body mass index and Katz Daily Living Activities Scale score (*p* < 0.001). A moderately positive and significant relationship was found between Successful Aging Scale score and WHO Well‐Being Index Scale score, Multidimensional Perceived Social Support Scale score (*p* < 0.001). Those who engaged in mild and moderate physical activity and did not have depressive symptoms had a high successful aging score. However, intense physical activity did not affect successful aging score (*p* > 0.05) (Table 4).

**TABLE 4:** Clinical variables affecting the score of successful aging.

			Successful aging score	
Clinical variables			*r*	*p* values
The self‐neglect score			0.741^a^	**< 0.001**
Katz Daily Life Activities Scale			0.251^a^	**< 0.001**
WHO‐5 Well‐Being Index			0.389^a^	**< 0.001**
Multidimensional Perceived Social Support Scale			0.576^a^	**< 0.001**

		Mean (SD)	*t*	*p* values
Depressive symptoms	Yes	40.4 (8.4)	– 3.71^b^	**< 0.001**
	No	44.2 (8.4)		
Mild physical activity each week	Yes	46.0 (8.2)	6.67^b^	**< 0.001**
	No	40.0 (7.8)		
Moderate physical activity each week	Yes	45.9 (7.7)	2.25^b^	**0.025**
	No	42.7 (8.6)		
Vigorous physical activity each week	Yes	47.7 (7.9)	1.46^b^	0.145
	No	43.0 (8.5)		

^a^ Pearson correlation test.

^b^ Independent samples *t*‐test.

To identify the predictors of successful aging, a multiple linear regression analysis was performed, taking into account variables that were shown to be significant in pairwise comparisons. As a result of this analysis, it was found that 62% of the variance for the dependent variable of successful aging in a significant regression model (*F* (12,303) = 44.28; *p* < 0.001) was explained by the independent variables (Adjusted *R* ^2^ = 0.62). According to this analysis, age, self‐neglect level, and presence of psychosocial support were determined as predictors of successful aging (Table 5).

**TABLE 5:** Factors affecting successful aging according to multivariate linear regression.^a^

Variables	*B*	*β*	*t*	*p* values	95% CI for B
Age	– 0.21	– 0.155	– 4.25	< 0.001	– 0.3 to 0.11
Self‐neglect	2.41	0.563	11.39	< 0.001	1.99–2.82
Multidimensional Perceived Social Support Scale	0.18	0.249	5.88	< 0.001	0.13–0.25
Constant	26.06	—	5.09	< 0.001	15.98–36.13

*Notes: B*, unstandardized beta coefficient; *β*: standardized beta coefficient; CI: confidence interval. Adjusted *R* ^2^ = 0.62,

*F* (12,303) = 44.28, *p* < 0.001.

^a^ Dependent variable: successful aging score. Independent variable: age, education level, working status, mild and moderate physical activity status, body mass index, health perception and health screening status, depressive symptoms, self‐neglect score, Multidimensional Perceived Social Support Scale score, Katz Daily Living Activities Scale score, and WHO‐5 Well‐Being Index Score.

## 4 Discussion

Successful aging has become an important concept worldwide to describe the quality of aging. It is a multidimensional concept and the main focus is on how to extend functional years in a later life. Successful aging perception can be affected by many personal, socioeconomic, and social factors. To the best of our knowledge, this is the first study to look into the connection between successful aging and self‐neglect among community‐dwelling older people, which can be a significant factor in successful aging. Results supported the hypothesis that self‐neglect was associated with successful aging. In addition, age and psychosocial support affect successful aging.

The average score of successful aging of the participants was 43.1 ± 8.5 (10–70 points). In two studies, the mean score of successful aging was 50.32 ± 17.42 and 60.2 ± 6.5 (Işık, Tekin, and Çağaltay Kayaoğlu 2021; Kütmeç Yılmaz 2020). Successful aging score is lower than the studies in the literature. Considering that the data were collected during the period of the pandemic, the successful aging score may have decreased due to the increase in inflation the weakening of the economic situation, the increase in social isolation, and the decrease in family support during the COVID‐19 period (Lee et al. 2021; McKinlay, Fancourt, and Burton 2021). Studies show that the perception of health status and quality of life of older people decreased (Özpınar et al. 2022) and the prevalence of social isolation and loneliness increased during the pandemic process (Su et al. 2023). Older people experienced some problems such as anxiety, depression, poor sleep quality, and physical inactivity during the isolation period (Sepúlveda‐Loyola et al. 2020). Interventions to protect the psychosocial health of community‐dwelling older people and promote physical activity should be planned against future crises since the rise in physical, mental, and social problems during the pandemic period severely impacts successful aging.

The self‐neglect mean score of the participants was moderate (6.6 ± 2.0 in 0–11 scores). However, it was determined that more than half of the participants neglected themselves. In a study conducted in Turkey, the self‐neglect score was 7.7 ± 2. In the same study, it was determined that 16.8% of older adults neglected themselves and older adults who exhibited self‐neglect had lower functionality, worse quality of life, and depressive symptoms than those who did not (İlhan et al. 2020). Another study reported that older people had a low self‐neglect score (94.5 ± 20.9, 60–300 scores) (Cicek, Sahin, and Erkal 2023). In South Korea and China, 23% of older people living alone frequently or always experienced self‐neglect (Lee and Kim 2014; Yu et al. 2019). Compared to the studies conducted before the pandemic process in the literature, the self‐neglect score in the present study is low, as is the successful aging score. This may be due to the restrictions during the pandemic period and the isolation of individuals at home, which may have caused them to neglect themselves. Older people should be re‐evaluated in terms of self‐neglect after the pandemic. There is a need for initiatives to prevent self‐neglect in case of any crisis such as the COVID‐19 pandemic in the future.

There is a strong positive relationship between the successful aging score and the level of self‐neglect. In regression analysis, self‐neglect is an important predictor of successful aging. Although there is no descriptive study evaluating the effect of self‐neglect on successful aging, there is a relationship between the two variables. Self‐neglect is an important variable for successful aging (İlhan et al. 2020). In a population‐based cohort study, self‐neglect and quality of life among older people may be an important component of successful aging (Dong 2014). Self‐neglect is thought to be an important factor for successful aging. The concept of self‐neglect, which refers to an older adult's incapacity or refusal to address their basic requirements, has strong reciprocal roles with successful aging. Therefore, there is a need for both qualitative and quantitative studies on this concept in the future. Additionally, as the pandemic process is over, there is a need to re‐evaluate the level of successful aging and self‐neglect.

Age, self‐neglect, and presence of psychosocial support were determined as the most important factors for successful aging. In a study in Malaysia, they reported that the factors affecting successful aging were young age, high education level and high income level, and ethnic origin (Hamid, Momtaz, and Ibrahim 2012). In a study in Brazil according to the logistic regression analysis, successful aging scores were 10.5 times higher for those without morbidity and 4.3 times for those aged 65–74 years, 2.7 times for those aged 75–84 years, 3.5 times for those with less than two chronic diseases, and 2.7 times for those with good daily living activities. Also in the same study, those with high social participation (2.1 times), those without depressive symptoms (2.1 times), those with high physical activity levels (1.9 times), and those who were overweight (obese) were found to be higher the successful aging scores (Canêdo et al. 2018). In a study in Turkey, the successful aging status of married people living with their spouses was better in Turkey (Kütmeç Yılmaz 2020). The health status, personal skills, income level, strong social relations with friends, family, neighbors, and social environment affect successful aging in Iran (Pashaki et al. 2015). Our findings were similar to the literature, and young‐old persons (65–74 years), those who do not neglect themselves, and the presence of psychosocial support are vital factors for successful aging. Thus, it may be helpful to develop interventional programs to prevent self‐neglect and strengthen psychosocial support in order to promote successful aging in individuals aged 75 years and over, who neglect themselves and those with insufficient psychosocial support.

## 5 Conclusion

The present study concluded that successful aging and self‐neglect scores were low, and self‐neglect was an important factor in successful aging. Moreover, successful aging was negatively impacted by becoming older and a lack of psychosocial support. Both self‐neglect and successful aging among community‐dwelling older people should be re‐evaluated after the pandemic. Successful aging has many dimensions and it is not always possible for every older person to reach all dimensions. It is thought that minimizing the level of self‐neglect in every older person will contribute to successful aging. Therefore, there is a need for more studies in the literature evaluating the effect of self‐neglect on successful aging. In addition, it is crucial for older people to be skilled enough to take care of themselves, provide the necessary self‐care, and age successfully. For this reason, it is recommended to conduct studies evaluating the effect of interventions to reduce self‐neglect on successful aging. Furthermore, it is crucial to concentrate on initiatives and programs that enhance the psychological support networks of older adults and promote healthy aging in individuals aged 75 years and over.

## 6 Limitations

This study has some limitations. Data were collected during the pandemic process and individuals who did not come to the FHC could not be included in the study. The study was only carried out at a FHC. It should be taken into consideration that since the study was carried out during the ongoing period of the pandemic, the selection of the target group could not be made by a random sampling method.

## Author Contributions

**Esma Nur Sert**: conceptualization, investigation, methodology, data curation, writing, original draft, formal analysis. **Aysegul Ilgaz**: conceptualization, investigation, methodology, supervision, formal analysis, writing, review and editing.

## Ethics Statement

The study was approved by the University of Akdeniz, Clinical Research Ethics Committee in Antalya, Turkey (number: KAEK‐674, date: 15.09.2021).

## Conflicts of Interest

The authors declare no conflicts of interest.

## Data Availability

The quantitative data used to support the findings of this study are available from the corresponding author upon request. Since the database was prepared in the author's own language.
